# Role of Kv7 channels in responses of the pulmonary circulation to hypoxia

**DOI:** 10.1152/ajplung.00362.2013

**Published:** 2014-10-31

**Authors:** Vojtech Sedivy, Shreena Joshi, Youssef Ghaly, Roman Mizera, Marie Zaloudikova, Sean Brennan, Jana Novotna, Jan Herget, Alison M. Gurney

**Affiliations:** ^1^Department of Physiology, Charles University - Second Faculty of Medicine, Prague, Czech Republic;; ^2^Faculty of Life Sciences, University of Manchester, Manchester, United Kingdom;; ^3^Department of Pathophysiology, Charles University - Second Faculty of Medicine, Prague, Czech Republic;; ^4^Department of Paediatrics, Charles University - Second Faculty of Medicine and University Hospital Motol, Prague, Czech Republic; and; ^5^Department of Biochemistry, Charles University - Second Faculty of Medicine, Prague, Czech Republic

**Keywords:** KCNQ, Kv7 channels, flupirtine, isolated lungs, hypoxic pulmonary vasoconstriction, P/Q relationship

## Abstract

Hypoxic pulmonary vasoconstriction (HPV) is a beneficial mechanism that diverts blood from hypoxic alveoli to better ventilated areas of the lung, but breathing hypoxic air causes the pulmonary circulation to become hypertensive. Responses to airway hypoxia are associated with depolarization of smooth muscle cells in the pulmonary arteries and reduced activity of K^+^ channels. As Kv7 channels have been proposed to play a key role in regulating the smooth muscle membrane potential, we investigated their involvement in the development of HPV and hypoxia-induced pulmonary hypertension. Vascular effects of the selective Kv7 blocker, linopirdine, and Kv7 activator, flupirtine, were investigated in isolated, saline-perfused lungs from rats maintained for 3–5 days in an isobaric hypoxic chamber (Fi_O_2__ = 0.1) or room air. Linopirdine increased vascular resistance in lungs from normoxic, but not hypoxic rats. This effect was associated with reduced mRNA expression of the Kv7.4 channel α-subunit in hypoxic arteries, whereas Kv7.1 and Kv7.5 were unaffected. Flupirtine had no effect in normoxic lungs but reduced vascular resistance in hypoxic lungs. Moreover, oral dosing with flupirtine (30 mg/kg/day) prevented short-term in vivo hypoxia from increasing pulmonary vascular resistance and sensitizing the arteries to acute hypoxia. These findings suggest a protective role for Kv7.4 channels in the pulmonary circulation, limiting its reactivity to pressor agents and preventing hypoxia-induced pulmonary hypertension. They also provide further support for the therapeutic potential of Kv7 activators in pulmonary vascular disease.

hypoxic pulmonary vasoconstriction (HPV) is an important physiological mechanism that helps to match ventilation with perfusion in the lungs. It reduces perfusion in poorly ventilated alveoli in order to optimize the oxygenation of arterial blood. The main sensors of O_2_ and effectors of HPV are pulmonary artery smooth muscle cells (PASMC) (30). The cellular mechanisms leading to HPV remain unclear, but the response probably involves multiple pathways that raise the cytoplasmic Ca^2+^ concentration ([Ca^2+^]_i_) and sensitize the smooth muscle contractile proteins to Ca^2+^ (50).

A consistent finding is that hypoxia depolarizes PASMC, at least in part by inhibiting K^+^ channels (29, 39, 43). The extent to which this contributes to HPV is controversial, opinions ranging from essentially no role (46) to its being a key initiator of HPV (3, 32, 34). Whether or not depolarization is required for HPV, it is clear that it has the potential to promote or enhance the response. With a sufficiently large depolarization, the membrane potential will reach the threshold for L-type Ca^2+^ channel activation (5), causing Ca^2+^ influx and promoting vasoconstriction. The nature of the K^+^ channels contributing to hypoxia-induced depolarization is also debated. Voltage-gated Kv1.5 and Kv2.1/9.3 channels have both been implicated (2–3, 32), as have voltage-independent, two-pore domain TASK channels (12, 38). Kv7 channels are new potential candidates, because several genes encoding Kv7 channel α-subunits (KCNQ1, KCNQ4, and KCNQ5) are expressed in PASMC and the channels appear to be active at the resting membrane potential (24, 25). This was demonstrated by the selective Kv7 channel inhibitors, linopirdine and XE991, causing pulmonary selective, endothelium-independent, but Ca^2+^-influx dependent vasoconstriction (24). The low voltage threshold for activation of Kv7 channels and their lack of inactivation during sustained depolarization suit Kv7 channels to a role in regulating the resting membrane potential (13). The susceptibility of Kv7 channels to pharmacological manipulation additionally makes them an attractive therapeutic drug target.

Following prolonged exposure to a hypoxic environment, for example in obstructive lung disease patients or at high altitude, the pulmonary circulation becomes hypertensive. This disease state is associated with sustained depolarization of the PASMC, along with loss of K^+^ channel activity (39, 47, 49, 55). The loss of K^+^ channel expression occurs early during the development of hypoxia-induced pulmonary hypertension (HPH), suggesting a causative role. Kv1.2, Kv1.5, and Kv2.1 were found to be downregulated within 24 h of exposure to hypoxia, while other K^+^ channel α- and β-subunits were unaffected (21). The expression of Kv7 channels in HPH has not been investigated, but in a mouse model of pulmonary hypertension (PH) induced by overexpression of the serotonin transporter, the ability of a Kv7 inhibitor to constrict and a Kv7 activator to dilate pulmonary arteries was markedly suppressed (35). Reduced vasodilation in response to Kv7 activators was also observed in systemic arteries from spontaneously hypertensive rats, where it was linked to loss of expression of the Kv7.4 channel subunit (23). Interestingly, despite the reduced sensitivity of pulmonary arteries to Kv7 modulators in mice with PH, the Kv7 activator, flupirtine, was able to return pulmonary artery pressure to normal, and it was able to prevent the development of HPH in mice exposed chronically to hypoxia (35).

This study investigated the role of Kv7 channel activity in the reaction of the rat pulmonary vascular bed to acute and short-term (3–5 days) hypoxia, in isolated, saline-perfused lungs. This preparation develops large pressor responses to airways hypoxia, but only if the lungs are first prestimulated, or primed, with a vasoactive substance that raises the basal perfusion pressure and overall vasoreactivity (16, 18, 33). The mechanism responsible for this priming effect is unknown, but it can be induced by the nonspecific Kv channel inhibitor 4-aminopyridine (4-AP) and other agents promoting smooth muscle depolarization and Ca^2+^ influx (15, 33). The ability of the Kv7 blocker, linopirdine, to modulate HPV was therefore investigated in both priming of the lungs to hypoxia and the steady-state HPV in preprimed, saline-perfused lungs. To assess the potential involvement of Kv7 channels in the development of HPH, we investigated how in vivo exposure to hypoxia for 3–5 days affects Kv7 channel expression and the responsiveness of the pulmonary circulation to Kv7 modulators. This period corresponds to the earliest time that structural changes and a rise in mean pulmonary artery pressure can be detected (19, 44). The mechanisms investigated may differ from those operating in established PH but are likely to be important during the onset of disease. In each case pulmonary vascular resistance, which is elevated in hypoxic rats (7), was measured from the slope of the pressure-flow (P/Q) relationship. Oral dosing with flupirtine assessed its ability to prevent early changes in pulmonary vascular reactivity that take place during the development of HPH in rats.

## METHODS

Experiments employed male Wistar rats aged 1–2 mo, weighing 290 ± 10 g (Biotest, Konarovice, Czech Republic) and were approved by the Animal Studies Committee at Charles University Second Medical School, in accordance with European Community and US National Institutes of Health guidelines for using experimental animals. Experiments on isolated lungs and in vivo exposure to hypoxia (normobaric chamber, Fi_O_2__ 0.1) were carried out in Prague. Arteries from lungs excised in Prague were either snap-frozen in liquid nitrogen for later protein extraction, stored in RNAlater (Life Technologies, Paisley, UK) for mRNA analysis, or mounted in paraffin blocks for sectioning and immunolabeling. Samples were shipped to Manchester for analysis.

### 

#### Isolated saline perfused rat lungs.

Isolated perfused rat lungs were prepared as described previously (17). Rats were anaesthetized (50 mg/kg ip thiopental) and ventilated through a tracheal cannula [peak inspiratory pressure 10 cmH_2_O, positive end-expiratory pressure (PEEP) 2 cm H_2_O, 50 breaths/min]. The chest was opened, heparin introduced into the right ventricle, and the pulmonary artery and left ventricle cannulated. The heart-lung block was placed into a humidified chamber and maintained at 38°C. The lungs were ventilated with a normoxic gas mixture, containing 21% O_2_ and 5% CO_2_, balanced with N_2_. The lung circulation was perfused with a physiological salt solution (PSS) containing albumin (4 g/100 ml perfusate) via the pulmonary artery, using a peristaltic pump. The perfusate dropped freely from the left ventricle cannula into a reservoir, from which it was pumped again into the pulmonary artery. PSS contained in mM: 119 NaCl, 4.7 KCl, 1.16 MgSO_4_, 17 NaHCO_3_, 1.18 KH_2_PO_4_, 3.2 CaCl_2_, and 5.5 d-glucose. The potentially confounding effects of endothelial vasoactive mediators like nitric oxide (NO) and prostaglandins were prevented by including inhibitors of their synthesis [17 μM meclofenamate and 50 μM nitro-l-arginine-methyl-ester (l-NAME)] in the perfusate. Although meclofenamate activates neuronal Kv7 channels comprising KCNQ2/3 subunits with an EC_50_ of 20 μM (40), it was reported to have no effect on vasoconstriction induced by the linopirdine analog XE991 (54). Meclofenamate is not therefore expected to interfere with the vascular action of linopirdine in isolated lungs. On the other hand, because meclofenamate can dilate isolated arteries, possibly as a consequence of activating Kv7 channels (54), we omitted this drug from the perfusate when testing the effects of the Kv7 activator, flupirtine.

#### Isolated lung protocols.

When investigating the effects of linopirdine on the pulmonary perfusion pressure and its response to stimulation, we perfused lungs at a constant flow rate of 4 ml·min^−1^·100 g^−1^. Changes in perfusion pressure therefore directly reflected changes in vascular resistance. The relationship between pulmonary perfusion pressure and perfusion flow (P/Q plot) was measured by increasing flow in a stepwise manner until it reached ∼150% of the basal level. Lungs were not ventilated during this protocol and the alveolar pressure was 2 cmH_2_O (PEEP). Before beginning P/Q measurements the lungs were perfused with PSS for 15 min to ensure a stable perfusion pressure, followed by priming with two cycles of angiotensin II application (0.2 μg added into the inflow cannula) followed by ventilation for 7 min with a hypoxic gas mixture (0% O_2_, 5% CO_2_, 95% N_2_) to induce HPV. After linopirdine or flupirtine was added to the perfusate the drugs could not be washed out to test for recovery. We therefore investigated drug effects by comparing groups of untreated lungs with treated lungs.

The effect of linopirdine on the responsiveness of the pulmonary circulation to hypoxia was first investigated in lungs that had been continually perfused with PSS for various periods but not primed with any vasoconstrictor stimulus. Linopirdine (230 μg) was administered as a bolus injection into the inflow cannula at the start of the experiment to give an effective concentration of ∼12 μM. This concentration was maximally effective at constricting rat pulmonary arteries in vitro (24) and within the range of EC_50_ values reported for Kv7 channel inhibition (45), while having little effect on a wide range of other ion channels (27, 36, 52, 53). Hypoxic pressor responses were elicited after 15, 30, and 65 min of lung perfusion by switching the ventilation gas mixture. HPV was then compared between lungs exposed to linopirdine (*n* = 6) and untreated, control lungs (*n* = 5).

The effects of linopirdine on HPV were also investigated in lungs that had been equilibrated for 15 min then primed by two cycles of angiotensin II (0.2 μg) injection followed by 7 min exposure to hypoxia. In this series of experiments we also investigated the effect of adding 4-AP, a nonspecific but mainly Kv1 channel blocker, in the presence of linopirdine. After priming, linopirdine was added to the reservoir to give a circulating concentration of 12 μM. After allowing 10 min to reach a steady state, we repeated stimulation with angiotensin II followed by hypoxia. In a separate group of lungs, linopirdine exposure was followed 10 min later by the addition of 4-AP to the reservoir, to give a circulating concentration of 3 mM, and after another 10 min the lungs were challenged again with angiotensin II followed by hypoxia. The perfusion pressures before and during the test stimulation with angiotensin II or hypoxia were measured and compared before and after the lungs were treated with linopirdine only or linopirdine followed by 4-AP.

The effects of flupirtine were tested on isolated lungs that had been primed by two cycles of angiotensin II followed by acute airways hypoxia. Flupirtine was added to the reservoir to give a circulating concentration of 20 μM. At this concentration flupirtine evokes nearly 50% of its maximum pulmonary vasodilator effect (25) and activates Kv7 channels, while having minimal effects on a number of other ion channels (26). Higher concentrations were not tested, because even at 20 μM, flupirtine caused partial inhibition of Ca^2+^ channel currents in bladder smooth muscle cells (1).

#### In vivo treatment.

This part of the study was designed to investigate the in vivo effects of the Kv7 activator flupirtine on hypoxic pulmonary hypertension induced by ventilatory hypoxia. Groups of rats were exposed to an hypoxic environment by maintaining them in an isobaric hypoxic chamber (Fi_O_2__ 0.1) for 5 days (14). An age-matched control group of rats was kept in room air (normoxia, *n* = 6). One group of rats exposed to hypoxia was administered flupirtine 15 mg/kg twice a day by gavage (*n* = 6) throughout the exposure period. As flupirtine was dissolved in dimethyl sulfoxide (DMSO), a further group exposed to hypoxia was administered the same volume of DMSO as a vehicle control (*n* = 6). A third group (hypoxia control) was exposed to hypoxia but received no other treatment (*n* = 6). At the end of the treatment period, isolated perfused lungs were prepared as above for subsequent in vitro experiments.

#### mRNA analysis.

As many intrapulmonary arteries as possible were dissected from rat lungs and used for the extraction of total RNA with an RNeasy Micro Kit (Qiagen). Real-time quantitative PCR was performed on cDNA synthesized from the DNase-treated RNA. Primers were designed with Gene Runner software (version 3, Hasting software) and Vector NTI (Invitrogen) for KCNQ1, KCNQ4 and KCNQ5, using GenBank sequences with the respective accession numbers NM_0320773, XM_233477, and XM_237012. Where possible, primers were designed to span introns, to detect any contamination by genomic DNA. The primer sequences are listed in Table 1. Reactions were carried out in 25 μl volumes containing 1 μl cDNA, 12.5 μl SYBR Green master mix, 10 μl H_2_O, and 7.5 pmol of each primer, using an Applied BioSystems 7500 PCR system, according to the manufacturer's instructions. Amplicons were 77–106 bp long. The cycling parameters were 95°C for 15 min, followed by 40 cycles at 95°C for 1 min, 58°C for 40 s, and 68°C for 40 s. A dissociation step was performed at the end of the reaction for melting curve analysis, a single peak in the curve representing specific production of the product. ABI 7500 software was used for data analysis. An absolute quantification method was used, in which we determined the input copy number by relating the PCR signal to a standard curve. Expression levels were then normalized against the housekeeping gene glyceraldehyde-3-phosphate dehydrogenase, measured simultaneously in the same samples using the primers listed in Table 1. Experiments were carried out in triplicate from the pooled RNA of three rats exposed to normoxia or hypoxia.

**Table 1.** Primers used for quantitative PCR analysis of KCNQ expression

Gene	GenBank Accession Number	Primer Pair Sequences (5′-3′) Forward; Reverse	Span Region
KCNQ1	NM_032073	GGCTCTGGGTTTGCACTG;	1131–1236
		CATAGCACCTCCATGCAGTC	
KCNQ4	XM_233477	CCCCGCTGCTCTACTGAG;	1181–1266
		ATGACATCATCCACCGTGAG	
KCNQ5	NM_001134643	CGAGACAACGACAGATGACC;	2012–2088
		TGGATTCAATGGATTGTACCTG	
GAPDH	NM_017008	CCATCAAGGACCCCTTCATT;	164–343
		CACCAGCATCACCCCATTT	

#### Protein expression.

Kv7.4 protein expression was measured in arteries from control rats, rats exposed to hypoxia for 4 days, and rats administered flupirtine (30 mg/kg/day) for 1 day before and during exposure to hypoxia for 4 days (*n* = 4). As many arteries as possible were collected from each lung and homogenized with a Wheaton glass tissue grinder (VWR International, Lutterworth, UK) in ice-cold RIPA buffer, containing 25 mM Tris·HCl (pH 7.5), 150 mM NaCl, 1% NP-40, 0.5% sodium deoxycholate, 1 mM ethylenediaminetetraacetic acid (EDTA), 1% sodium dodecyl sulfate (SDS), and 1× cOmplete, Mini, EDTA-free Protease Inhibitor Cocktail (Roche Diagnostics, Burgess Hill, UK). Samples were centrifuged at 1,000 *g* for 2 min, and we analyzed the supernatant by Western blotting after determining the total protein concentration using a BCA protein assay kit (Thermo Fisher Scientific, Cramlington, UK). Supernatant samples were incubated for 7 min at 95°C with Laemmli loading buffer containing 25 mM Tris·HCl (pH 6.8), 10% glycerol, 5% β-mercaptoethanol, 2% SDS, and 0.04% bromophenol blue. Proteins were separated by 10% SDS-PAGE, transferred onto a Immobilon-P PVDF membrane [Millipore (UK), Watford, UK] and washed three times in Tris-buffered saline (TBS: 25 mM Tris·HCl, 150 mM NaCl, pH 7.3) with 0.1% Tween 20 (TTBS). The membrane was blocked for 1 h at room temperature with 5% milk powder in TTBS then cut between the 50 and 70 kDa size markers. The upper part of the membrane was incubated overnight at 4°C with a mouse monoclonal anti-Kv7.4 antibody (cat. #75-082, Neuromab) diluted in 1% milk powder in TTBS. The lower part was treated in the same way, but with an antibody directed against β-tubulin (Sigma, Poole, UK) as an internal control. After being washed, the membranes were then incubated for 2 h with a horseradish peroxidase-conjugated secondary antibody (Jackson ImmunoResearch Laboratories, West Grove, PA), diluted in 1% milk powder in TTBS. Antibody binding was detected with SuperSignal West Femto (Kv7.4) or Pico (β-tubulin) Chemiluminescent Substrate kits (Thermo Fisher Scientific) and a ChemiDoc imaging system (Bio-Rad).

We validated the Kv7.4 antibody by comparing Western blots obtained from proteins extracted from wild-type human embryonic kidney 293T (HEK-293T) cells and HEK-293T cells transfected with recombinant KCNQ4 (GenBank accession number AF105202) using the pcDNA 3.1 Expression Vector (Life Technologies) and X-tremeGENE9 (Roche Diagnostics).

#### Immunostaining.

Lungs were removed en bloc, perfused via the trachea (12 Torr) and pulmonary artery (25 Torr) with 4% paraformaldehyde and then dipped in paraformaldehyde for 24 h. The left lung was cut into four sections and fixed in paraformaldehyde for 4 days before being washed in running water for 3–4 h. The fixed sections were then dehydrated with alcohol in increasing concentration: 80% for 24 h, 96% for 4 h, and then absolute alcohol overnight. After being dipped in cedar oil for 2 days, the sections were incubated in xylene for 10–15 min and then embedded in paraffin wax. We deparaffinized issue sections (5 μm) cut with a microtome and rehydrated by dipping them in xylene and graded alcohol as follows: xylene for 5 min twice, 100% alcohol for 3 min twice, 90% alcohol for 3 min, 70% alcohol for 3 min, phosphate-buffered saline (PBS) in distilled H_2_O for 3 min twice. The sections were placed in citrate buffer and heated in a microwave at medium power for 15 min before being washed three times with PBS and permeabilized with 0.1% Triton X-100 in PBS for 1 h. After being blocked with 1% BSA for 1 h, tissue sections were incubated with an anti-Kv7.4 antibody (Santa Cruz, S18, 1:100 dilution) for 24 h and then probed for 1 h with secondary antibody conjugated to Alexa fluor 594 (Molecular Probes). The fluorescent DNA marker 4′,6-diamidino-2-phenylindole was added at 2 μg/ml to enable visualization of cell nuclei. Duplicate sections were processed without primary antibody or after the primary antibody was incubated with excess antigen, for controls. Fluorescence was imaged using a confocal microscope with ×40 water dipping objective (Nikon).

#### Analysis of plasma NO.

Blood samples were collected from the left ventricles of rats used to study the in vivo effects of flupirtine, before the lungs were prepared for experiments. Plasma was separated from the blood, and the total plasma concentration of NO and its oxidation products (NOx) was measured with a NO chemiluminescence analyzer (Sievers model 280i) as previously described (20, 22).

#### Drugs.

Linopirdine dihydrochloride and flupirtine maleate were purchased from Tocris Bioscience and prepared as 10 mM stock solutions dissolved, respectively, in water or DMSO. Aliquots of the stock solutions were stored frozen and thawed once for each experiment. All other drugs were from Sigma Aldrich. 4-AP was dissolved in PSS and 0.2 ml added to 40 ml of circulating perfusate for each lung preparation.

#### Statistical analysis.

All data are shown as means ± SE and analyzed by Statview software with ANOVA or repeated-measures ANOVA and Fisher's protected least significant difference post hoc test. Where indicated a paired *t*-test was used. Differences were considered statistically significant when *P* < 0.05.

## RESULTS

### 

#### Linopirdine primes HPV in saline-perfused rat lungs.

**Fig. 1.**
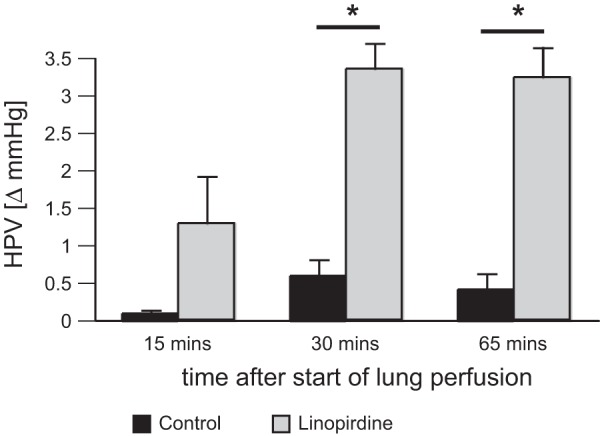
Linopirdine primes hypoxic pulmonary vasoconstriction (HPV) in saline-perfused rat lungs. HPV response measured in unprimed lungs 15, 30, or 65 min after bolus injection of 230 μg linopirdine into the inflow cannula to give an effective concentration of ∼12 μM (gray, *n* = 6) and in time-matched controls (black, *n* = 5). *P < 0.05 vs. control.

In the absence of priming, salt-perfused lungs respond poorly to hypoxia (33), and this was seen in our study (Fig. 1). The basal perfusion pressure before each challenge with hypoxia was not found to differ significantly at any time point between the control and linopirdine-treated lungs. There was also no significant change in the basal perfusion pressure during the experiments. At 15 min it was 10 ± 1.5 mmHg in control lungs and 8.1 ± 0.57 mmHg in lungs exposed to linopirdine. The values at 30 min and 65 min were respectively (control vs. linopirdine) 9 ± 1.1 vs 7.89 ± 0.46 mmHg and 9 ± 1.1 vs 8.0 ± 0.52 mmHg. Thus, in the absence of priming, pulmonary perfusion pressure was unaffected by linopirdine. In contrast, the pressor response to acute hypoxia was found to be significantly potentiated, by approximately sevenfold, at 30 and 65 min after linopirdine injection (Fig. 1).

#### Linopirdine potentiates HPV in primed lungs.

**Fig. 2.**
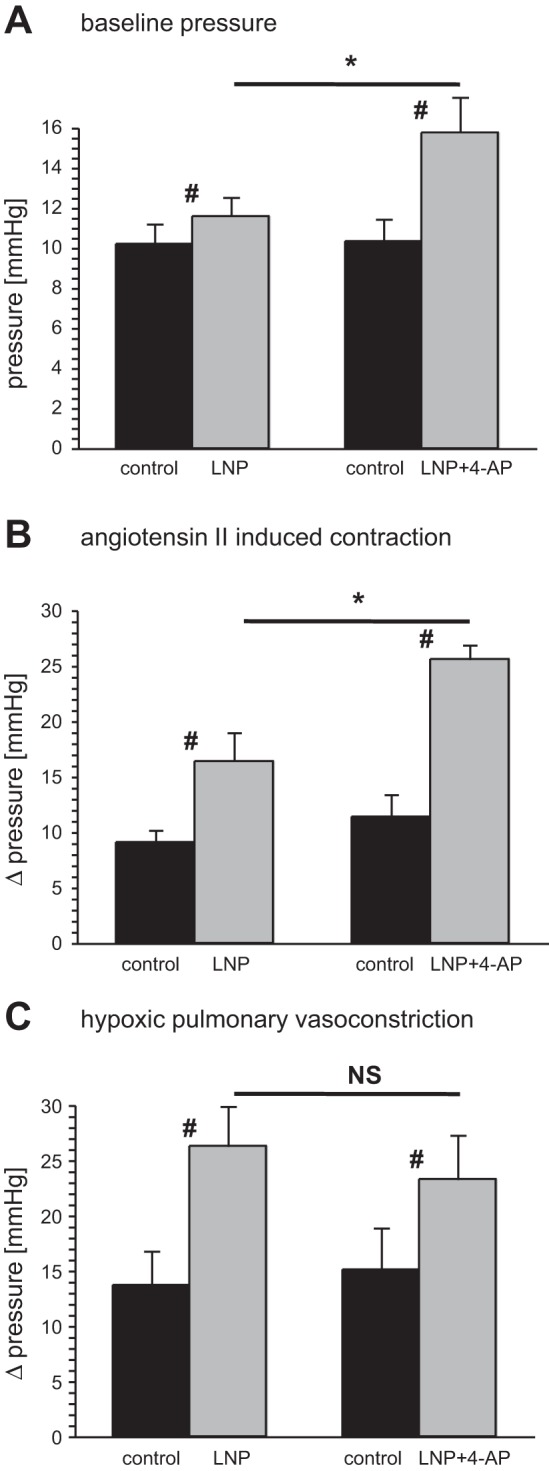
Kv channel inhibition modulates pulmonary vascular responses to hypoxia and angiotensin II in primed lungs. Baseline perfusion pressure (*A*), angiotensin II-induced vasoconstriction (*B*), and HPV (*C*) measured in primed lungs before (control, black bars) and after exposure to 12 μM linopirdine (LNP) or 12 μM linopirdine plus 3 mM 4-aminopyridine (4-AP) (LNP + 4-AP) (gray bars). #*P* < 0.05 control vs. LNP or LNP + 4-AP, *P < 0.05 LNP vs. LNP + 4-AP; *n* = 6 for both group. NS, not significant.

The effects of linopirdine on primed lungs are summarized in Fig. 2. Before Kv channel blockers were administered, the basal perfusion pressure and the reactivity to angiotensin II and hypoxia did not differ between the groups at any time point. The administration of linopirdine to primed lungs, either on its own or with 4-AP, caused an increase in basal perfusion pressure (Fig. 2*A*), reflecting its vasoconstrictor action. The rise in pressure caused by linopirdine alone was the same in both groups: 1.4 ± 0.24 mmHg (*n* = 6) in lungs exposed to linopirdine only and 2.0 ± 0.7 mmHg (*n* = 6) in lungs that were later exposed to 4-AP as well. The addition of 4-AP caused a further increase in perfusion pressure, of 4.3 ± 0.37 mmHg over and above that induced by linopirdine.

The pressor response to angiotensin II in primed lungs was enhanced by linopirdine, and it was further enhanced when 4-AP was added (Fig. 2*B*). In contrast, although HPV was potentiated by the Kv channel blockers, the addition of 4-AP did not cause any greater increase than that produced by linopirdine on its own (Fig. 2*C*). Linopirdine caused the pressor response to hypoxia to increase from 14 ± 3 to 26 ± 4 mmHg (*P* < 0.05, paired *t*-test). With the combined administration of linopirdine and 4-AP, the pressor response to hypoxia increased from 15 ± 4 to 23 ± 4 mmHg (paired *t*-test, *P* < 0.05), which was not significantly different from that seen with linopirdine alone.

#### Loss of Kv7 channel activity early in the development of HPH.

**Fig. 3.**
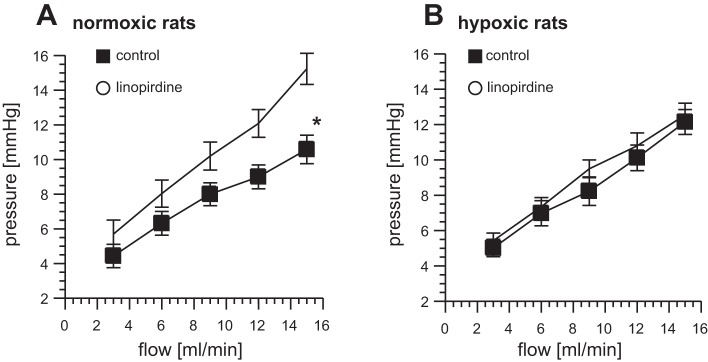
Loss of response to linopirdine in chronic hypoxia. Pressure-flow (P/Q) plots measured in primed lungs from normoxic (*A*) and 3-day hypoxic (*B*) rats in control conditions and after exposure to linopirdine (10 μM). *P < 0.05 linopirdine vs. control; *n* = 6 for each group.

The P/Q relationships measured during stepwise increases in flow rate were linear (R^2^ > 0.94) in all primed lungs studied, whether from rats exposed for 3–5 days to isobaric hypoxia or maintained in a normoxic environment. The slope of the line corresponds to incremental flow resistance and the pressure axis intercept to the average critical closing pressure (41, 48). The P/Q relationships measured in normoxic lungs (*n* = 6) and 3-day hypoxic lungs (*n* = 6) did not differ significantly (Fig. 3; compare normoxic control in *A* with hypoxic control in *B*). The ability of linopirdine to constrict pulmonary vessels in primed lungs was, however, lost in the hypoxic rats. Although linopirdine caused a significant elevation of the baseline pulmonary perfusion pressure in the lungs of rats exposed to normoxic air, it had no effect in the lungs from matched hypoxic rats. This difference is apparent in the P/Q relationships measured before and 10 min after the addition of linopirdine (10 μM) to the reservoir (Fig. 3): two-factor ANOVA indicates a significant effect in control, but not hypoxic lungs. Linopirdine increased the slope of the P/Q relationship in normoxic lungs from 0.49 ± 0.01 to 0.81 ± 0.08 mmHg·min/ml (*P* < 0.05, paired *t*-test), without changing the intercept with the pressure axis (Fig. 3*A*). Neither the slope nor the pressure intercept in hypoxic lungs were affected by linopirdine (Fig. 3*B*).

#### Effect of hypoxia on the dilator response to flupirtine.

**Fig. 4.**
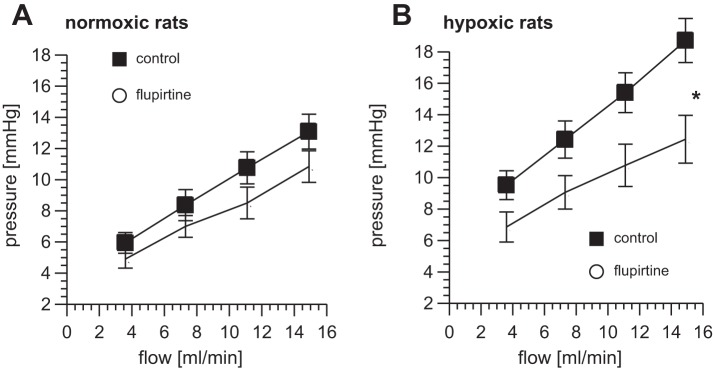
Flupirtine causes pulmonary vasodilatation in chronic hypoxia. Effect of flupirtine (20 μM) on P/Q plots measured in primed lungs from normoxic controls (*A*) and rats exposed for 5 days to hypoxia (*B*). *P < 0.05 flupirtine vs. control; *n* = 5 for each group.

In contrast to what was found with linopirdine, flupirtine, circulating at a concentration of 20 μM, had no effect on the pulmonary perfusion pressure of lungs from control rats but caused pulmonary vasodilation in the lungs from hypoxic rats. Figure 4 shows P/Q measurements (*n* = 5) made in primed lungs, with or without the addition of flupirtine to the reservoir 10 min after priming. Two-factor ANOVA indicates a significant effect of flupirtine only on the lungs from hypoxic rats. When the slope and pressure intercept of the P/Q plot were analyzed separately, flupirtine was found to have no significant effect on either parameter in the normoxic lungs. In contrast, the lungs from the hypoxic rats displayed a significant reduction in the slope of the P/Q relationship, without a change in the pressure intercept (Fig. 4*B*). The slope fell from 0.75 ± 0.07 mmHg·min/ml in control conditions to 0.49 ± 0.05 mmHg·min/ml (*P* < 0.05) after addition of flupirtine, indicating a drop in incremental flow resistance.

#### Altered expression of KCNQ4 mRNA in hypoxic pulmonary arteries.

**Fig. 5.**
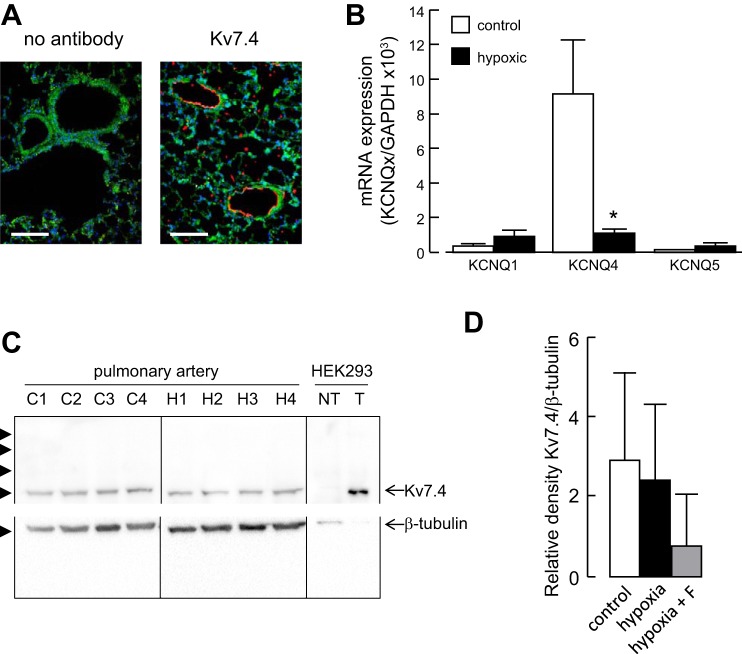
Hypoxia downregulates Kv7.4 mRNA expression. *A*: fluorescence images of lung sections from normoxic rats showing autofluorescence (green) and labeling with an anti-Kv7.4 antibody (red) and the nuclear marker 4′,6-diamidino-2-phenylindole (DAPI) (blue). Sections were treated identically, except for omission of the Kv7.4 antibody in the control. Calibration bars 100 μm. *B*: expression profile of KCNQ1, KCNQ4, and KCNQ5 subunit mRNAs in rat pulmonary artery from rats maintained in a normoxic (control) or hypoxic environment for 3 days. Detected with quantitative RT-PCR and normalized to the expression of GAPDH (*n* = 3). *P < 0.05 hypoxic vs. control. *C*: Western blots of pulmonary artery proteins from 5 separate normoxic (C1–C4) and hypoxic (H1–H4) rats and proteins from nontransfected HEK-293T cells (NT) and HEK-293T cells overexpressing Kv7.4 channels (T). Proteins were separated on a 10% SDS-PAGE and transferred to a PVDF membrane, which was cut between the 50 and 75 kDa markers and probed separately with antibodies against Kv7.4 and β-tubulin. Arrowheads indicate the positions of molecular weight markers (kDa). *D*: densitometric analysis of Western blots showing Kv7.4 expression normalized to β-tubulin in arteries from normoxic (control) and hypoxic rats, as well as rats administered flupirtine (F, 30 mg/kg/day) and exposed to hypoxia for 5 days (*n* = 4).

Immunostaining of fixed lung sections showed that Kv7.4-positive cells are mainly localized to blood vessels and form a ring around the blood vessel lumen (Fig. 5*A*). Figure 5*B* shows the relative expression of KCNQ1, KCNQ4, and KCNQ5 mRNAs in pulmonary arteries from age-matched rats maintained for 3 days in a hypoxic or normoxic environment. While no significant differences were detected in the expression of KCNQ1 or KCNQ5 mRNA between hypoxic and normoxic lungs, there was a significant loss of KCNQ4 mRNA expression in the hypoxic lungs. Western blots confirmed the expression Kv7.4 protein in pulmonary arteries from normoxic and hypoxic lungs (Fig. 5*C*). Densitometric analysis of the protein bands did not detect a significant change in Kv7.4 protein (measured relative to β-tubulin) in vessels from hypoxic rats, whether or not they were administered flupirtine (Fig. 5*D*).

#### Flupirtine inhibits hypoxic pulmonary hypertension.

Table 2 shows the effects of 5-day hypoxia and oral flupirtine treatment (30 mg/kg/day) on the P/Q relationship and vascular reactivity measured in isolated lungs. Five-day exposure to hypoxia caused an increase in the P/Q slope relative to the normoxic controls, indicative of increased incremental flow resistance. This increase was absent in the rats treated with flupirtine, but not in those treated with vehicle. Thus flupirtine prevented the early rise in pulmonary vascular resistance that leads to HPH. Exposure to hypoxia or flupirtine did not affect the P/Q intercept (Table 2). Moreover, lungs from animals exposed to 5-day hypoxia had an enhanced response to acute hypoxia compared with the normoxic controls. This effect of hypoxia was also prevented by flupirtine treatment but not vehicle. Flupirtine did not alter the overall reactivity of hypoxic lungs, because the vasoconstriction to angiotensin II was potentiated in all groups exposed to hypoxia (Table 2).

**Table 2.** Flupirtine treatment decreases incremental flow resistance and HPV in rats exposed to 5-day hypoxia

Group	P/Q Slope, mmHg·min/ml	P/Q Intercept, mmHg	HPV, Δ mmHg	ANG II Constriction, Δ mmHg	NOx in Plasma, μM
Normoxia	0.541 ± 0.052	3.6 ± 0.7	5.3 ± 1.0	5.6 ± 0.6	26.0 ± 2.5
Hypoxia	0.672 ± 0.05*	3.7 ± 0.4	8.5 ± 1.1*	9.3 ± 1.3*	46.6 ± 8.3*
Hypoxia + flupirtine	0.475 ± 0.022†	5.3 ± 0.5	4.9 ± 1.0†	11.7 ± 1.4*	47.5 ± 2.6*
Hypoxia + vehicle	0.678 ± 0.111*	3.6 ± 1.1	9.6 ± 1.4*	9.0 ± 0.8*	40.0 ± 2.5*

The pressure-flow (P/Q) slope and intercept, amplitude of hypoxic pulmonary vasoconstriction (HPV), and angiotensin II (ANG II) induced vasoconstriction and total plasma concentration of nitric oxide (NO) and its oxidation products (NOx) in rats exposed to normoxic or hypoxic conditions. One group of hypoxic rats also received 30 mg/kg/day flupirtine, while another group had an equivalent volume of vehicle.

* *P* < 0.05 vs. normoxia.

† *P* < 0.05 vs. hypoxia and hypoxia with vehicle; *n* = 6 for each group.

#### Plasma NO levels.

Kv7 activators dilate pulmonary arteries through a direct action on smooth muscle (25). Activators of K_ATP_ channels also dilate pulmonary arteries by hyperpolarizing the smooth muscle (4) and inhibit HPH (37). Recent studies suggest, however, that the effectiveness of K_ATP_ channel activators in HPH may be due to an action on endothelial K_ATP_ channels, which rescues NO production from the dysfunction induced by hypoxia (57). As Kv7 channel expression in endothelial cells has not been addressed, we tested the possible involvement of such an effect in the response to short-term in vivo hypoxia and the protective effect of flupirtine by measuring plasma levels of NO and its oxidation products (NOx). Table 2 shows that the plasma concentration of NOx was increased in all groups of rats exposed to hypoxia, but it was unaffected by flupirtine.

## DISCUSSION

The results of this study implicate Kv7 channels in the development of HPV and the response to short-term hypoxia in vivo. In saline-perfused lungs, the specific Kv7 channel blocker, linopirdine, was found to prime the lungs for HPV and to potentiate HPV after priming. Kv7 channels may therefore play an inhibitory role, hyperpolarizing the membrane and preventing excitation and Ca^2+^ influx. Exposing rats to hypoxia for a few days reduced the expression of KCNQ4 mRNA and the responsiveness of the pulmonary circulation to Kv7 modulating drugs. This suggests that there is loss of Kv7.4 channel activity, which would contribute to enhanced excitation and vasoreactivity. Despite the apparent loss of functional Kv7 channels, there appeared to be little change in the level of Kv7.4 protein expression, and flupirtine was able to return the raised pulmonary vascular resistance to control levels and to prevent the effects of short-term hypoxia. The lack of effect of flupirtine on plasma NO confirmed that its protective effect against hypoxia was due to its direct action on pulmonary artery smooth muscle and not to enhanced endothelial function.

HPV depends on the level of tone present in the pulmonary circulation at the time O_2_ is reduced (9, 33). Vascular tone is normally low (7), at least in part because of K^+^ channels that mediate a background K^+^ efflux from PASMC and drive the membrane potential to a negative value, thereby preventing voltage-gated Ca^2+^ channels from opening. While several distinct K^+^ channels have been proposed to contribute to this background K^+^ efflux, many lack the biophysical properties necessary to fulfill such a role effectively (11, 12, 39). Although less is known about Kv7 channels in the pulmonary circulation, their properties suggest that they would be active at the resting potential of PASMC and able to contribute to the background K^+^ efflux (13). These properties, characteristic of homo- or heteromeric Kv7 channels formed from the KCNQ1, 4, or 5 genes, include a low voltage threshold for activation (below −60 mV) and lack of inactivation during sustained depolarization (45).

We previously reported that blockade of Kv7 channels with linopirdine (0.5–10 μM) causes dose-dependent vasoconstriction in saline-perfused rat lungs (25), consistent with Kv7 channels being open and contributing to the PASMC resting potential. In that study, we tested linopirdine after priming the lungs with cycles of angiotensin II-hypoxia stimulation. We have now found that without priming, linopirdine (at 12 μM) does not cause a detectable change in the baseline pulmonary perfusion pressure. In this respect linopirdine behaves much like hypoxia, which only raises pulmonary artery pressure in saline-perfused lungs after they have been primed with a substance that enhances vasoreactivity (33). Interestingly, despite the lack of effect of linopirdine on the baseline perfusion pressure in unprimed lungs, it was able to prime the lungs for HPV, causing marked enhancement of the HPV response. The most likely explanation for this is that linopirdine depolarized the PASMC, thereby facilitating the effects of hypoxia, but the depolarization was too small by itself to activate sufficient Ca^2+^ influx for contraction (5).

Although HPV in saline-perfused lungs is known to require priming, why the effects of linopirdine should require priming is unclear. Both linopirdine and hypoxia depolarize myocytes by 10–15 mV (25, 39, 56). If the cells are in a hyperpolarized state before priming this may not be enough to open Ca^2+^ channels. Alternatively, vasodilator influences generated by the endothelium or other lung cells could offset any depolarization or stimulated Ca^2+^ influx. Although meclofenamate and l-NAME were included in the perfusate to prevent interference from prostaglandin and NO production (18), we cannot rule out influences from other substances generated in the salt-perfused lungs, either before or during priming. Priming may alternatively reflect changes in Ca^2+^ homeostasis, which in myocytes is regulated by complex coupling between ion channels and transporters in the plasmalemma, sarcoplasmic reticulum (SR), and mitochondria (28). During priming with repeated cycles of angiotensin II-hypoxia, the myocytes are stimulated to contract and raise pulmonary artery pressure. Underpinning the contractions are transient increases in [Ca^2+^]_i_, due to Ca^2+^ entry from the extracellular space and the SR. Ca^2+^ entering the cell further serves to replenish the SR store, in order to maintain contraction. Although Ca^2+^ enters the cell in resting conditions, it is rapidly buffered by the peripheral SR, from where it is returned to the extracellular space (28). Thus in the absence of stimulation it is possible that the central SR, required for contraction, becomes depleted of Ca^2+^ and priming serves to replenish the store. This could be important for the priming of HPV, which has been shown to depend on SR Ca^2+^ release (50), but it does not easily explain priming of the linopirdine response, which relies exclusively on Ca^2+^ entry (24). Perhaps the rise in [Ca^2+^]_i_ during priming activates Ca^2+^-dependent enzymes (28), which alter the activity of Kv7 channels, the activation threshold of Ca^2+^ channels, or the Ca^2+^ sensitivity of contraction. Another possible explanation is that activation of the Rho-kinase and/or protein kinase C pathways by angiotensin II during priming leads to persistent Ca^2+^ sensitization, which amplifies the contractile response to Ca^2+^ influx.

The increase in perfusion pressure induced by linopirdine in primed lungs was due to vasoconstriction and an increase in pulmonary vascular resistance, because it was accompanied by an increase in the slope of the P/Q relationship. This agrees with its vasoconstrictor effect on isolated pulmonary artery, where it was measured after routine priming with repeated exposure to KCl (24). The pressor response to linopirdine could be further potentiated by 4-AP. At the concentration tested (12 μM), linopirdine is maximally effective on isolated rat pulmonary arteries (24), and 3 mM 4-AP is expected to fully block 4-AP-sensitive Kv channels (6). The additive nature of their effects on pulmonary perfusion pressure is consistent with the drugs acting through independent mechanisms, most likely by inhibiting different ion channels to give an additive effect on membrane potential.

Linopirdine enhanced the pressor response to angiotensin II and further potentiated HPV after it was primed with angiotensin II. Both of these effects may reflect a larger [Ca^2+^]_i_ signal, caused by depolarization-induced Ca^2+^ influx adding to the sources of Ca^2+^ mobilized by hypoxia or angiotensin II. The potentiation of HPV was not due simply to the increase in baseline vascular tone, because increasing it further with 4-AP had no additional effect on HPV. On its own 4-AP is known to enhance HPV (15). The lack of synergy between 4-AP and linopirdine suggests that they potentiate HPV by the same mechanism, i.e., depolarization. This is consistent with the idea that sensitivity to hypoxia may be conferred by a “priming” depolarization that activates O_2_-sensitive Kv channels, which would normally oppose the depolarization and minimize Ca^2+^ influx but are inhibited by hypoxia (51). It may not matter how the depolarization is generated. The additive effects of linopirdine and 4-AP on the angiotensin II response suggest that the pressor responses to hypoxia and angiotensin II involve distinct mechanisms.

In rats exposed for only 3 days to a hypoxic environment, the vasoconstrictor effect of linopirdine was essentially abolished. This loss of activity correlated with markedly reduced expression of the KCNQ4 mRNA, but not KCNQ1 or 5. Thus it appears that the pulmonary pressor effect of linopirdine may require K^+^ channels containing the Kv7.4 subunit. The result also implicates Kv7.4 channel downregulation in the early phases of development of HPH, and this is likely to contribute to the PASMC depolarization seen around this time (21). Positive staining with a Kv7.4 antibody, seen as a distinct ring around blood vessels, is consistent with expression of the Kv7.4 protein in PASMC. Despite the apparent loss of Kv7 function and Kv7.4 mRNA, we did not detect a significant reduction in Kv7.4 protein in the arteries taken from hypoxic rats at the same time. Protein levels may take longer to fall than the mRNA. On the other hand, as protein levels were assessed in the whole blood vessel, changes in smooth muscle membrane protein may have been missed. Unfortunately, we could not extract sufficient protein to isolate the membrane fraction at detectable levels. The loss of Kv7 function could therefore have been caused by a loss of membrane protein. As the molecular chaperone heat shock protein 90 (Hsp90) is required for Kv7.4 channel assembly in the membrane (10), an impaired interaction between these proteins could also contribute. Three days' exposure to hypoxia was sufficient to disrupt the interaction of Hsp90 with endothelial nitric oxide synthase and impair NO-dependent pulmonary vasodilation in piglets (8).

The Kv7 activator, flupirtine, had little effect on the perfusion pressure recorded from primed normoxic lungs. This probably reflects the low basal pulmonary vascular tone in these lungs, because to see a dilator effect on rat isolated artery preparations it was necessary to preconstrict the vessels (25). Interestingly, despite the apparent loss of functional Kv7.4 channels in hypoxic lungs, flupirtine produced a drop in pulmonary perfusion pressure, due to reduced vascular resistance. The ability to evoke vasodilation probably reflects raised intrinsic tone in the hypoxic lungs, but the mechanism is less clear. One possibility is that the loss of expression of Kv7 function and/or another K^+^ channel (21) led to an increase in membrane resistance, thereby amplifying the hyperpolarization produced by activating a small number of Kv7 channels. Direct evidence for altered membrane resistance in response to hypoxia is lacking, but a decrease in resistance seems more likely (49). Although flupirtine could have produced its effects by activating residual Kv7.4 channels, activation of Kv7.5 channels or a heterologous combination of Kv7.4/7.5 is also possible. Flupirtine does not activate Kv7.1 channels (45), so although they are expressed in pulmonary artery, Kv7.1 channels could not mediate the drug's effects. Blockade of Ca^2+^ channels could also contribute to the dilator action of flupirtine, because both flupirtine (at 20 μM) and its structural analog retigabine have been reported to cause inhibition of Ca^2+^ channel currents in smooth muscle cells (31).

The ability of flupirtine to dilate vessels in hypoxic lungs suggests it may be able to reverse or counteract the depolarization caused by hypoxia, which promotes voltage-gated Ca^2+^ influx and vasoconstriction, as well as smooth muscle cell proliferation (42). The ability to restore the membrane potential to a normal level could have beneficial effects over and above those of calcium channel antagonists, because it would not only inhibit calcium influx but also restore the electrical driving force for a range of ions that cross the cell membrane. The beneficial effects of flupirtine found in this study mirror its effects in a mouse model of HPH (35). Along with the finding that flupirtine could reverse spontaneous PH in a further mouse model (35), these studies implicate Kv7 channels in the early development of HPH and suggest Kv7 activators should be explored further to determine their potential as a treatment for PH in patients.

## GRANTS

This work was supported by the Grant Agency of Czech Ministry of Health NT/13358, Czech Science Foundation 13-01710S (to J. Herget), and the British Heart Foundation (to A. M. Gurney).

## DISCLOSURES

No conflicts of interest, financial or otherwise, are declared by the author(s).

## AUTHOR CONTRIBUTIONS

V.S., S.J., M.Z., J.H., and A.M.G. conception and design of research; V.S., S.J., Y.G., R.M., S.B., and J.N. performed experiments; V.S., S.J., Y.G., R.M., S.B., and J.N. analyzed data; V.S., S.J., Y.G., R.M., M.Z., S.B., J.N., J.H., and A.M.G. interpreted results of experiments; V.S. and S.B. prepared figures; V.S., S.J., M.Z., J.H., and A.M.G. drafted manuscript; V.S., M.Z., J.H., and A.M.G. edited and revised manuscript; V.S., J.H., and A.M.G. approved final version of manuscript.
